# The Plasma Mitochondrial DNA Is an Independent Predictor for Post-Traumatic Systemic Inflammatory Response Syndrome

**DOI:** 10.1371/journal.pone.0072834

**Published:** 2013-08-20

**Authors:** Xiaoling Gu, Yanwen Yao, Guannan Wu, Tangfeng Lv, Liang Luo, Yong Song

**Affiliations:** 1 Department of Respiratory Medicine, Jinling Hospital, Nanjing University School of Medicine, Nanjing, China; 2 Intensive Care Unit, Wu xi Second Affiliated Hospital, Nanjing Medical University, Wu xi, China; University of Leicester, United Kingdom

## Abstract

**Background and Purpose:**

Mitochondrial DNA (mtDNA), a newly identified damage-associated molecular pattern, has been observed in trauma patients, however, little is known concerning the relationship between plasma mtDNA levels and concrete post-traumatic complications, particularly systemic inflammatory response syndrome (SIRS). The aim of this study is to determine whether plasma mtDNA levels are associated with injury severity and cloud predict post-traumatic SIRS in patients with acute traumatic injury.

**Patients and Methods:**

Eighty-six consecutive patients with acute traumatic injury were prospectively enrolled in this study. The plasma mtDNA concentration was measured by a real-time, quantitative PCR assay for the human ND2 gene. The study population’s clinical and laboratory data were analyzed.

**Results:**

The median plasma mtDNA was higher in trauma patients than in healthy controls (865.196 (251.042-2565.40)pg/ml vs 64.2147 (43.9049-80.6371)pg/ml, P<0.001) and was independently correlated with the ISS score (r=0.287, P<0.001). The plasma mtDNA concentration was also significantly higher in patients who developed post-traumatic SIRS than in patients who did not (1774.03 (564.870-10901.3)pg/ml vs 500.496 (145.415-1285.60)pg/ml, P<0.001). Multiple logistic regression analysis revealed that the plasma mtDNA was an independent predictors for post-traumatic SIRS (OR, 1.183 (95%CI, 1.015-1.379), *P*=0.032). Further ROC analysis demonstrated that a high plasma mtDNA level predicted post-traumatic SIRS with a sensitivity of 67% and a specificity of 76%, with a cut-off value of 1.3185 µg/ml being established, and the area under the ROC curves (AUC) was 0.725 (95% CI 0.613-0.837).

**Conclusions:**

Plasma mtDNA was an independent indictor with moderate discriminative power to predict the risk of post-traumatic SIRS.

## Introduction

Trauma is a global public health problem and is the leading cause of death among people who are below 45 years of age. Twenty percent of these trauma-related deaths occurred several days to weeks after an injury and were mostly due to post-traumatic complications [1]. Systemic inflammatory response syndrome (SIRS), a catastrophic inflammatory response, is a potentially life-threatening trauma-related complication that is observed in up to 30% of trauma patients and is characterized by increases in cellular [2] and non-cellular proinflammatory agents [3]. Although the potential of anti-inflammatory agents for modifying inflammatory processes has been clearly established, large-scale clinical studies investigating anti-inflammatory interventions using such modulators have frequently shown little benefit in terms of patient survival [4,5,6]. The failure of these trials has been attributed partially to the current inability to accurately identify high-risk patients at an early stage after injury [7]. Therefore, at present, it continues to be urgent to identify new biomarkers that would aid in the accurate early stratification of populations at high risk for post-traumatic complications, particularly SIRS.

In the last ten years, there has been considerable interest in the use of circulating plasma mitochondrial DNA (mtDNA) as a potential marker in various diseases. Plasma mtDNA can be defined as mtDNA fragments that are detectable in circulating plasma. Previous publications have demonstrated that plasma mtDNA could be detected in normal healthy populations, albeit at notably low baseline levels [8]. Simultaneously, elevated levels of plasma mtDNA have been observed in patients with a variety of critical conditions, including malignancy [9,10], acute ischemic stroke [11], severe sepsis [12], corrosive injury [13], and trauma [8]. Lam’s research group [8] reported that plasma mtDNA increased soon after trauma and was positively correlated with the severity of injury severity. Additionally, a series of studies demonstrated that mtDNA could be recognized by pattern recognition receptors and trigger immune inflammatory responses. Recently, Zhang [14] demonstrated that it is likely that plasma mtDNA that is released into circulation after shock acts in a damage-associated molecular pattern, activating neutrophils and inducing an inflammatory phenotype, ultimately contributing to the initiation of SIRS. Considering the proinflammatory potential of circulating plasma mtDNA, we hypothesised that the circulating plasma mtDNA levels is probably related to the development of post-traumatic inflammation-related complications and has the potential to serve as a predictive indictor for post-traumatic complications, such as SIRS, organ failure and acute lung injury.

Although previous studies have demonstrated a significant increase in the level of circulating plasma mtDNA after trauma and that these mtDNA levels are positively associated with the severity of an injury [8], little is known concerning the relationship between plasma mtDNA levels and concrete post-traumatic complications. Therefore, in the present study, we measured the plasma mtDNA levels on admission in both post-traumatic SIRS present patients and post-traumatic SIRS absent patients, and conducted a stepwise logistic regression analysis to investigate the significance of elevated plasma mtDNA levels on admission in terms of its potential to predict post-traumatic complications, particularly SIRS.

## Patients and Methods

### Ethics Statement

The present study protocol was approved by Jingling Hospital’s Institutional Review Committee on Human Research. All patients and healthy adult volunteers provided written informed consent before any study-related procedure was performed.

### Study population and definition

The present study investigating the potential of higher levels of plasma mtDNA in predicting post-traumatic SIRS was conducted in an 11-month period (December 2011 to October 2012). Eighty-six eligible patients at Jingling hospital in Nanjing were recruited. All of these patients were older than 18 and younger than 80 years of age and had been admitted to the emergency ICU department because of an acute traumatic injury. The exclusion criteria included pregnancy, malignant disease, acute ischemic stroke, sepsis, drug overdose, shock with hypoperfusion, chronic renal failure, liver cirrhosis, SIRS at admission and those who with a history of surgery within the preceding three months. For comparison, forty healthy adult volunteers who received a physical examination and had no history of trauma or evidence of infection were recruited as controls.

### Clinical assessment and data collection

We prospectively recorded patients’ medical records according to the pre-existing standardized evaluation forms, which included the patients’ demographic data, Acute Physiology and Chronic Health Evaluation (APACHE) II score on admission [15], and the Injury Severity Score (ISS) at the time of discharge [16]. Basic laboratory tests, in particular a routine blood assay, were performed on admission. Additionally, all eligible patients were screened daily for SIRS according to specific criteria. Patients were diagnosed with SIRS if they had two or more of the following concurrent conditions: (a) a body temperature <36 °C or >38 °C, (b) a heart rate >90/min, (c) a respiratory rate >20/min or presence on the ventilator at PCO_2_<32 mmHg, (d) a white blood cell count (WBC) <4000/mm^3^ or >12000/mm^3^. No standardized treatment protocol was used in the present study because of its nature as observational research.

### Blood sampling and assessment of plasma mitochondrial DNA

Blood samples were collected within 2 hours after admission to the emergency ICU department. Under minimal tourniquet pressure, a 2-ml blood sample was collected from the antecubital vein with a 19-gauge needle and syringe upon patient presentation. These blood samples were collected into EDTA-containing tubes and prepared for further mtDNA extraction. Another sample that would be used for basic laboratory tests was collected according to routine clinical blood collection procedures.

To obtain cell-free plasma, EDTA-blood samples were initially centrifuged at 3000 rpm (900 g) for 10 min, and the plasma was transferred into a clear polypropylene tube with great care not to disturb the buffy coat layer. Next, the newly separated plasma was centrifuged at 10000 rpm(9600 g) for another 10 min, and the upper portion of the plasma was removed into another clear tube and stored at -80 °C prior to DNA extraction. The plasma fraction was separated as soon as possible after sample collection. DNA in the plasma was isolated with the QIAamp DNA Blood Kit (Qiagen) according to the ‘blood and body fluid protocol’ provided by the manufacturer. We used a 200-µl plasma sample for each extraction, and the extracted DNA was eluted with 50 µl double-distilled water. The A260/280 ratio of palsma DNA was 1.7 to 2.0, excluding any significant protein contamination. Blood samples from 40 controls were also processed in the same manner.

The amount of mtDNA was measured with MT-ND2 genes by a real-time quantitative PCR assay that was performed on a 7300 real time PCR system (Applied Biosystems). The MT-ND2 gene was present specifically in the mitochondrial genome. The MT-ND2 PCR system consisted of 1.6 µl DNA solution, 10 µl FastStart Universal SYBR Green ROX Mix(2X) (Roch), and 300 nmol/L of each of the amplification primers MT-ND2-156F (5’-CAC AGA AGC TGC CAT CAA GTA-3’) and MT-ND2-245R (5’-CCG GAG AGT ATA TTG TTG AAG AG-3’), which has been previously described by Kung [12] and cloud detect a 90-base-pair amplicon. The final reaction volume was 20 µl. The thermal profile was set up as follows: a first denaturation step at 95°C for 10 min followed by 40 cycles at 95°C for 15S, 60°C for 25S and 72°C for 30S. The generation of a plasma mtDNA standard curve with a range from 3.0 to 3.0*10^^-5^µg/ml was accomplished using purified mtDNA, which was extracted from cultured THP-1 cells and quantified with the absorbance at 260 nm. Each sample was run in triplicate, and the mean value was used for further calculations. The quantitative results of the mtDNA were expressed as pg/ml. The investigators performing these measurements were blind to the patients’ clinical information.

### Statistical analysis

Data were expressed as the median (inter-quartile ranges, IQR), absolute numbers or percentages. Continuous variables were compared using the nonparametric Mann-Whitney test, while categorical variables were analyzed using Fisher’s exact test. The Spearman-rank correlation was used to explore the relationships among age, ISS score, APACHE II score, both the length of ICU and hospital stay, C-reactive protein (CRP), WBC, neutrophil-to-lymphocyte ratio (NLR) and levels of the trauma patient’s plasma mtDNA. Variables associated significantly with the concentration of plasma mtDNA were further tested by multiple linear regression analysis, while levels of plasma mtDNA were logarithmically transformed to be used as the dependent variable. A stepwise logistic regression analysis was performed to identify factors that had independent predictive value for the risk of post-traumatic SIRS. Furthermore, to determine the predictive power of independent factors, we constructed receiver operator characteristics (ROC) curves and calculated the areas under the curve (AUCs) with 95% CIs. The optimal cut-off values were selected to obtain the optimal compromise between specificity and sensitivity. In all of the abovementioned tests, *P*<0.05 was considered to be statistically significant. All statistical analyses were performed using SPSS 17.0 software.

## Results

### Patient Characteristics

Eighty-six adults (61 males and 25 females; age 18-80 years) with traumatic injuries were recruited into the study. Thirty-six of the 86 (41.86%) patients were diagnosed with post-traumatic SIRS during the course of the disease. The baseline characteristics of the study patients are presented in Table 1. The majority of patients were involved in motor vehicle accidents or Pedestrian struck, and 13 of the 86 patients (15.1%) had a pre-injury illness, in particular, hypertension and diabetes mellitus. The median APACHE II score and ISS score was 9 (7–12) and 18 (13–22), respectively. None of the patients died in the ICU or in the hospital. The median length of stay in the ICU was 4 (1.5-7.5) days, and the median time of the days of hospitalization was 12 (9–16) days. With the study patients stratified by post-traumatic complications (SIRS absent/SIRS present), the APACHE II score, ISS score, and the length of ICU or hospital stay were significantly higher in the patients who developed post-traumatic SIRS compared with patients who did not develop post-traumatic SIRS (11.5 (8–16) vs 9 (6–11), *P*=0.004; 22 (18–29) vs 14 (9.75-18.25), *P* <0.001; 7 (4-10.5) vs 2 (1–4), *P* <0.001; 14.5 (11–18) vs 9.5 (7–13), *P* <0.001). There were no significant differences in the age or the proportion of male patients between these two groups.

**Table 1:** Baseline characteristics of patients in the study.

**Characteristics**	**SIRS absent**	**SIRS present**	**Total**
No. of patients	50	36	86
Age, years	47 （30.25-57.25)	44 （26-57.75)	45.5 (28.75-57.25)
Male sex	33 (66)	28 (77.8)	61 (70.1)
Mechanism of injury			
Motor vehicle accident	24 (48.0)	20 (55.6)	44 (51.2)
Pedestrian struck	13 (26.0)	10 (27.8)	23 (26.7)
Fall	8 (16.0)	5 (13.9)	13 (15.1)
Assault	5 (10.0)	1 (2.8)	6 (7.0)
Comorbidities of patients prior to trauma			
Diabetes Mellitus	2 (4.0)	1 (2.8)	3 (3.5)
Hypertension	11 (22.0)	2 (5.6)	13 (15.1)
APACHE II score	9 (6-11)	11.5 (8-16)	9 (7-12)
ISS score	14 (9.75-18.25)	22 (18-29)	18 (13-22)
Ventilatory Treatment	1 (2.0)	5 (13.9)	6 (7.0)
Length of ICU stay, days	2 (1-4)	7 (4-10.75)	4 (1.5-7.5)
Length of hospital stay, days	9.5 (7-13)	14.5 (11-18)	12 (9-16)
Plasma mtDNA, pg/ml	500.496 (145.415-1285.60)	1774.03 (564.870-10901.3)	865.196 (251.042-2565.40)

Data are median (IQR) or n (%).

Abbreviations: mtDNA, mitochondrial DNA.

### Plasma mtDNA levels in trauma patients

**Figure 1:**
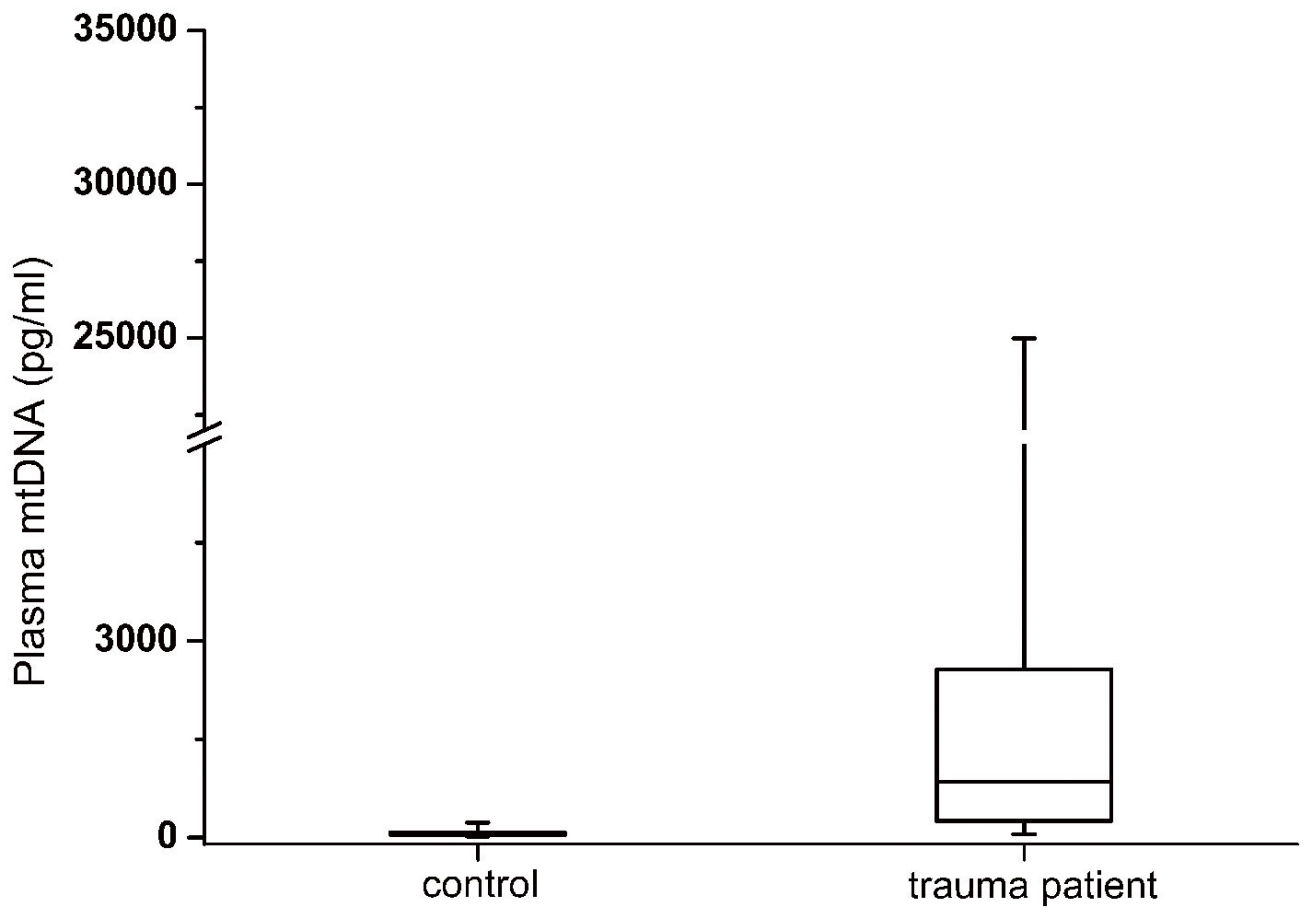
Box plots of the plasma mitochondrial DNA concentration between the trauma patients and healthy controls. Plasma mtDNA concentrations presented as median values (line across the boxes), IQR (limits of the boxes), and 5^th^ to 95^th^ percentiles (whiskers). The plasma mtDNA concentration in the traumatic patients was significantly higher than for the healthy controls (P<0.001).

**Figure 2:**
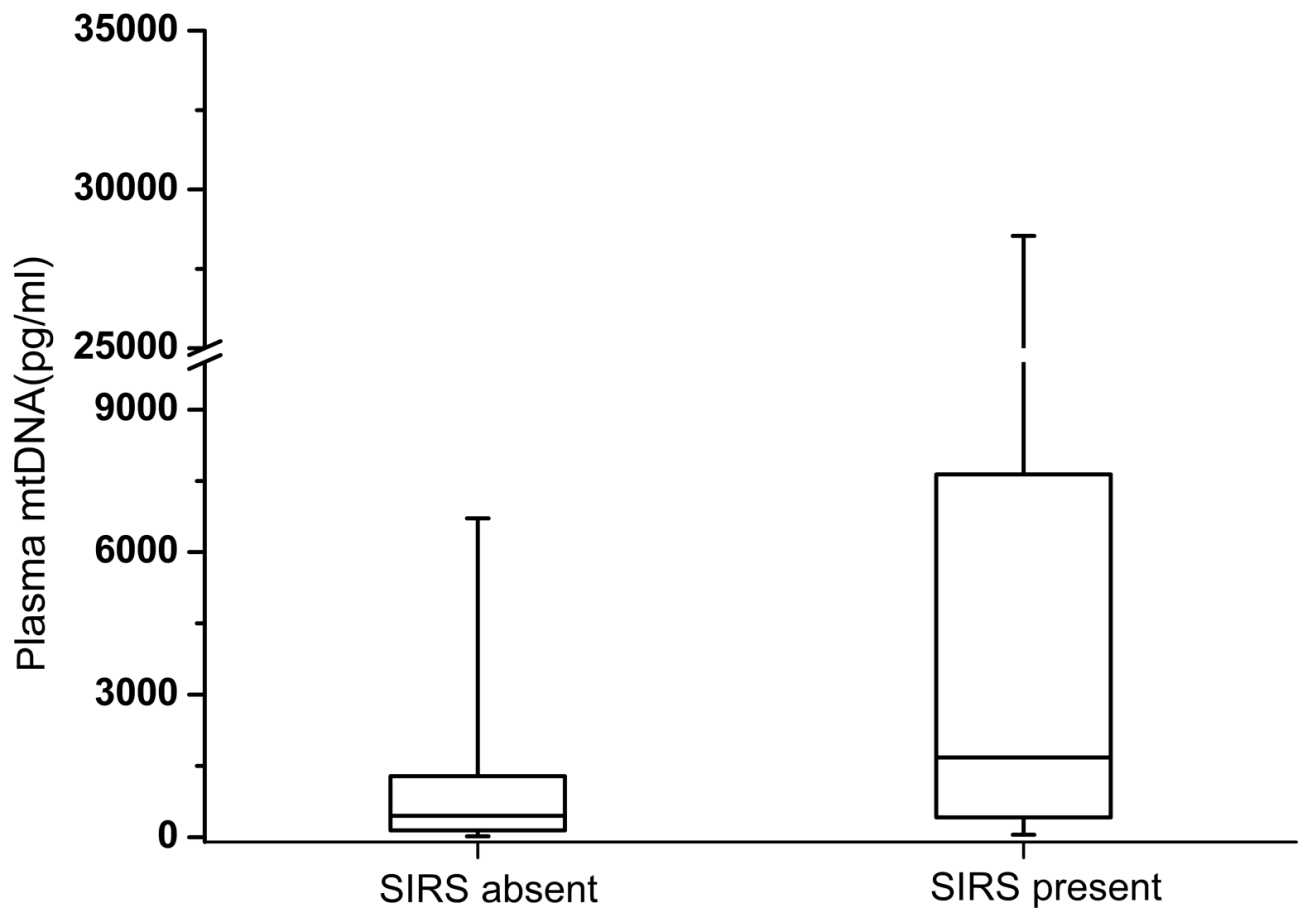
Box plots of the plasma mitochondrial DNA concentration in patients stratified by post-traumatic systemic inflammatory response syndrome present or absent. Plasma mtDNA concentrations presented as median values (line across the boxes), IQR (limits of the boxes), and 5^th^ to 95^th^ percentiles (whiskers). The plasma mtDNA concentration in patients who developed post-traumatic SIRS was significantly higher than for those who did not (P<0.001).

The median plasma mtDNA concentration in the trauma patients was 865.196 (251.042-2565.40)pg/ml, which was significantly higher than that observed in the controls (*P* <0.001, Figure 1). The median plasma mtDNA level was also significantly higher in the patients who developed post-traumatic SIRS compared with patients who did not develop post-traumatic SIRS (1774.03 (564.870-10901.3)pg/ml vs 500.496 (145.415-1285.60)pg/ml, *P*<0.001, Figure 2). According to the SIRS scores, patients with post-trauma SIRS were further divided into mild SIRS subgroup (SIRS score=2, n=24) and moderate/severe SIRS subgroup (SIRS score≥3, n=12). The results of statistical analyses demonstrated that the median concentration of plasma mtDNA in the moderate/severe subgroup was significantly higher than that in the mild subgroup (9819.47 vs 1509.93 pg/ml, P=0.012). This data indicated that the plasma levels of mtDNA on admission were associated with the severity of post-trauma SIRS. Additionally, among all enrolled eighty-six trauma patients, eight patients (10.52%) later developed into organ dysfunction, including one patient diagnosed as multiple organ failure. The median concentration of plasma mtDNA in the patients with and without organ dysfunction was 2205.50 and 672.822 pg/ml, respectively. The difference were statistically significant (p=0.048).

### Correlation of plasma mtDNA levels and clinical severity, as well as laboratory data

**Figure 3:**
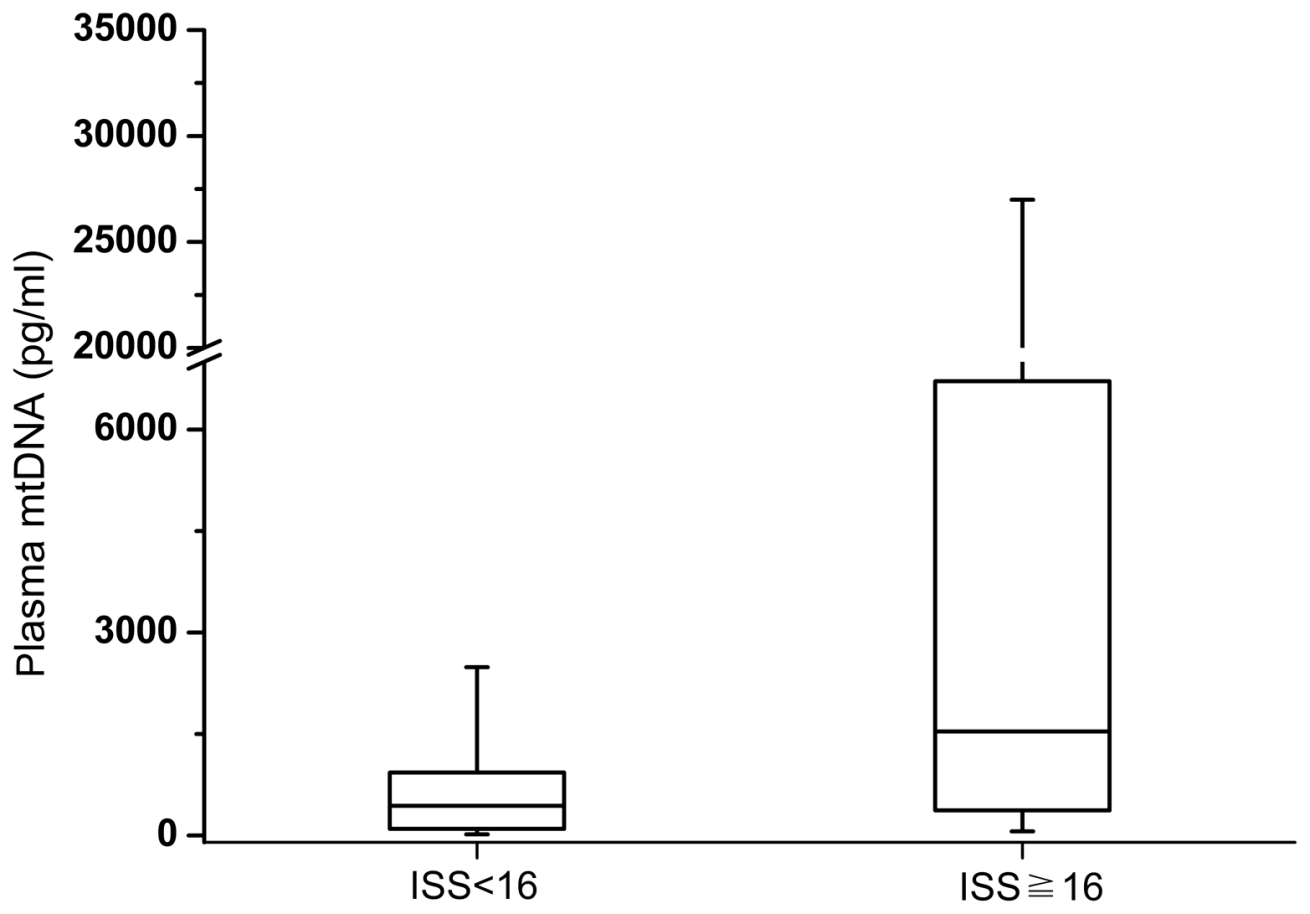
Box plots of plasma mitochondrial DNA concentration in trauma patients stratified by the severity of injury. Plasma mtDNA concentrations presented as median values (line across the boxes), IQR (limits of the boxes), and 5^th^ to 95^th^ percentiles (whiskers). Plasma mtDNA concentrations in traumatic patients with ISS ≧ 16 were significantly higher than in patients with ISS < 16 (P<0.001).

By correlation analysis, the median plasma mtDNA level significantly correlated with the APACHE II score (r=0.230, *P* =0.034), the ISS score (r=0.454, *P* <0.001), and the NLR (r=0.319, *P* =0.003). However, there were no statistical correlations between plasma mtDNA and WBC (r=0.199, *P* =0.068), or between plasma mtDNA and CRP (r=0.126, *P* =0.252). Additionally, neither age nor sex was associated with the plasma mtDNA concentration (*P* =0.330 and *P* =0.213, respectively). A further multiple linear regression analysis demonstrated that the ISS score was independently positively correlated with the plasma mtDNA concentration (r=0.287, *P* <0.001). The median concentrations of plasma mtDNA in the minor/moderate (ISS <16) injury subgroup and the severely (ISS≧16) injured subgroup were 439.866 (104.037-1003.32)pg/ml and 1628.21 (722.943-3040.21)pg/ml, respectively. The difference between these two groups was statistically significant (*P*=0.001, Figure 3).

### Predictive ability of plasma mtDNA

Thirty-six of the 86 (41.86%) patients were diagnosed with post-traumatic SIRS during the course of the disease. The median diagnosis time was 2 days after admission. The statistical assessment of the laboratory parameters on admission between SIRS-absent and SIRS-present patients revealed that all of the following conditions were significant: the WBC (*P*<0.001), CRP (*P*<0.001), NLR (*P*<0.001), and plasma mtDNA concentration (*P*<0.001). In the post-traumatic SIRS subgroup, 55.6% (20/36) of the patients had a WBC >10*10^9^/L (upper normal cutoff), and 94.4% (34/36) of the patients exhibited an abnormally high circulating CRP concentration that was higher than the ceiling value of the normal reference range. If the 95th percentile (0.3096 µg/ml) of the plasma mtDNA concentration in the healthy control group was defined as the upper normal cutoff value, 80.6% (29/36) of the patients in the post-traumatic SIRS subgroup exhibited an abnormally increased level of plasma mtDNA. Thus, there were significantly more patients with an abnormally increased plasma mtDNA than the patients with an abnormal WBC (*P*=0.042), which was almost equal to the number of patients with raised CRPs (*P*=0.151). This result indirectly indicated that similar to CRP, the changes in the plasma mtDNA level was observed sooner than the changes in the WBC count and that it would be appropriate to use plasma mtDNA as an early predictor of post-traumatic SIRS.

To compare various factors further, we conducted a logistic regression analysis to determine the factors with an independent predictive value for post-traumatic SIRS. Among the factors used in the logistic regression (i.e., age, sex, APACHEII score, plasma mtDNA concentration, WBC, CRP, and NLR), three indicators, including plasma mtDNA, WBC and CRP, were independently predictive of the risk of post-traumatic SIRS (Table 2).

**Table 2:** Independent predictors of post-traumatic systemic inflammatory response syndrome according to the results of logistic regression analysis.

**Variables**	**Odds Ratio (95%CI)**	***P* value**
mtDNA	1.183 (1.015-1.397)	0.032
CRP	1.022 (1.001-1.043)	0.036
WBC	1.886 (1.350-2.634)	<0.001

Abbreviations: mtDNA, mitochondrial DNA; CRP, C-reactive protein; WBC, white blood cell count; CI, confidence interval.

**Figure 4:**
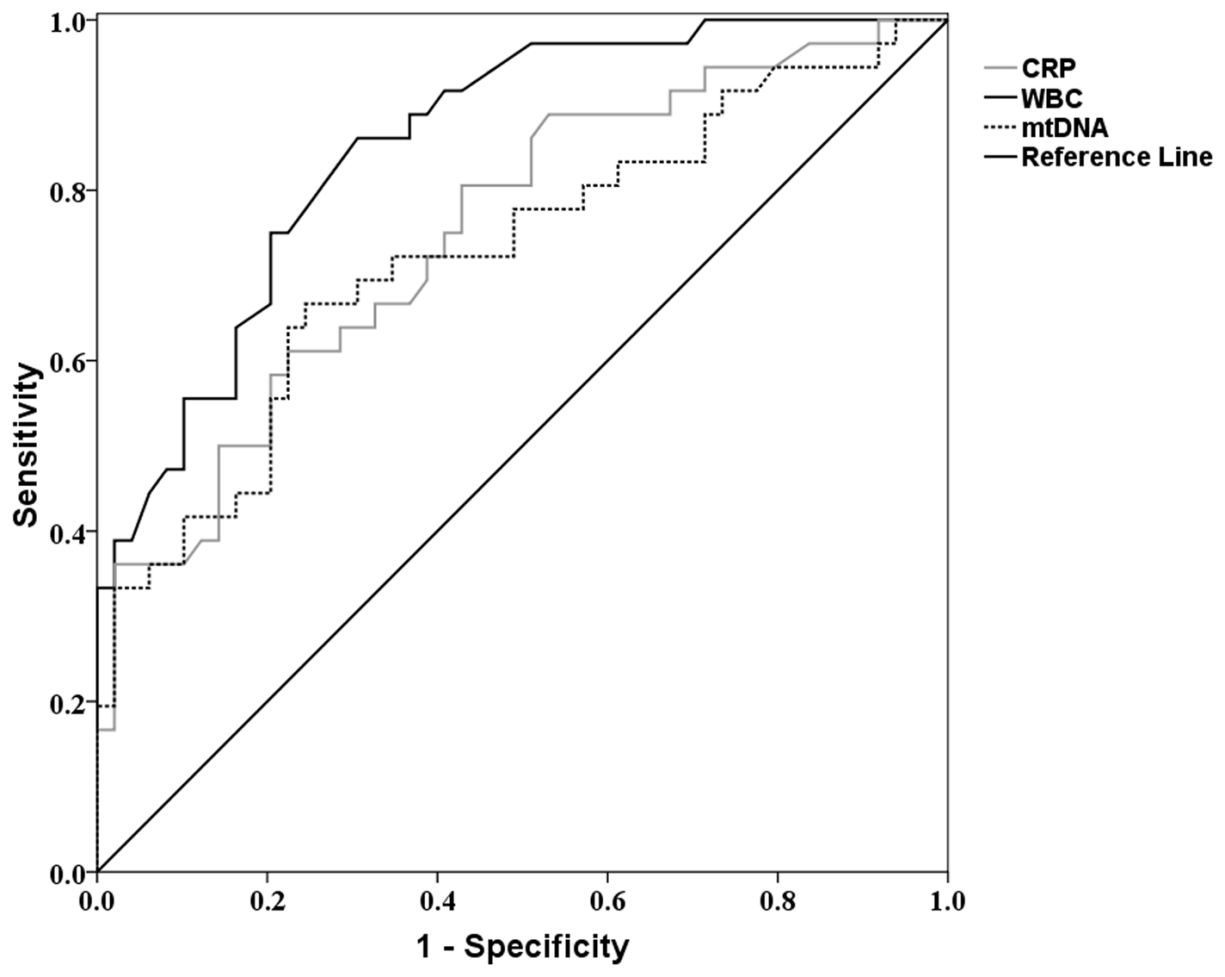
Receiver operator characteristics curves for plasma mitochondrial DNA levels, C-reactive protein concentrations and white blood cell count in predicting post-traumatic SIRS. The AUCs for plasma mitochondrial DNA, C-reactive protein and white blood cell count was 0.725(95%CI 0.613-0.837), 0.751(95%CI 0.646-0.855) and 0.853(95%CI 0.775-0.932), respectively.

Furthermore, ROC curves were calculated for these independent predictors as they were used in predicting the risk of post-traumatic SIRS (Figure 4). To predict the risk of post-traumatic SIRS, the area under the ROC curves (AUC) for plasma mtDNA was 0.725 (95% CI 0.613-0.837). The AUCs in the prediction of post-traumatic SIRS were slightly higher for WBC and CRP (0.853 (95%CI 0.775-0.932); 0.751 (95%CI 0.646-0.855), respectively). This finding indicated that although plasma mtDNA might be a less favorable predictor than CRP or WBC, it still had moderate discriminatory power for the prediction of post-traumatic SIRS. The optimal cutoff value of plasma mtDNA for the risk of post-traumatic SIRS was 1.3185 µg/ml with a sensitivity of 67% and a specificity of 76%.

## Discussion

To the best of our knowledge, the present study is the first to investigate the potential power of plasma mtDNA levels on admission as a predictor of post-traumatic complications, particularly SIRS. Previous studies have demonstrated that the plasma mtDNA concentration was increased significantly after trauma [8,17] and that the elevated concentrations were positively correlated with injury severity. Plasma mtDNA may be a promising marker for the prediction of trauma-related mortality, as the median level of plasma mtDNA was significantly higher in trauma patients who later died compared with that observed in the survivors [8]. However, these previous studies provided little information about the relationship between the plasma mtDNA concentration and concrete post-traumatic complications. In this study, we demonstrated the significance of plasma mtDNA in predicting the risk of post-traumatic SIRS.

This study primarily assessed plasma mtDNA concentration in acute traumatic patients and produced four major findings. First, the median concentration of plasma mtDNA was significantly higher in trauma patients than in healthy controls and was independently correlated with injury severity, as assessed by the ISS score. Second, the plasma mtDNA concentration was positively correlated with markers of systemic inflammatory response in terms of NLR. Third, the plasma mtDNA level on admission was significantly higher in patients who developed post-traumatic SIRS compared with patients who did not. Additionally, the increase in plasma mtDNA was observed sooner than the changes in the WBC; thus, it would be more appropriate to use plasma mtDNA as an early predictor of post-traumatic SIRS. A further logistic regression analysis demonstrated that plasma mtDNA was one of three independent predictors for post-traumatic SIRS. Finally, the ROC analysis revealed that the plasma mtDNA concentration had a moderate discriminatory power to predict the risk of post-traumatic SIRS.

In the present study, plasma mtDNA was defined as the mtDNA fragments that can be detected in circulating plasma. Although the actual source of plasma mtDNA has remained elusive, previous publications have demonstrated that the time course of the changing pattern of plasma mtDNA is similar to that of nuclear DNA in various critical conditions including acute ischemic stroke [11], severe sepsis [12], and trauma [8]. It is likely that this similarity indicates that plasma mtDNA might have the same origin as nuclear DNA, which has been suggested to be involved with either necrosis or apoptosis [18]. Thus, the plasma mtDNA would be theoretically correlated with tissue necrosis caused by the force of trauma and may even be a marker of an injury’s severity. Moreover, Zhang [17] has reported that severe trauma may lead to a rapid release of mtDNA into circulation, and Lam’s group [8] additionally demonstrated that there was a positive correlation between plasma mtDNA levels and ISS score. Consistent with these results, our study demonstrated that the median concentration of plasma mtDNA in severely (ISS≧16) injured subgroup was significantly higher than in the minor/moderate (ISS <16) injury subgroup. Furthermore, we conducted multiple linear regression analysis and determined that the plasma mtDNA level was independently positively correlated with the severity of injury that was reflected in the ISS score.

Additionally, the molecular components of mtDNA are similar to those of bacterial DNA, which consists of inflammatogenic unmethylated CpG motifs [19,20]. A series of recent studies also demonstrated that mtDNA could be recognized by pattern recognition receptors and trigger immune inflammatory responses. Studies by Oka [21] demonstrated that mtDNA that escaped from autophagy-mediated degradation in cardiomyocytes would trigger an inflammatory response similar to the inflammation generated by bacterial DNA. Moreover, Shimada [22] reported that oxidized mtDNA released during apoptosis could activate the NLRP3 inflammasome and induce the secretion of caspase-1-dependent proinflammatory cytokines. The above two studies indicated that mtDNA could precipitate the activation of innate immunity in the original cell. In actuality, mtDNA also can initiate systemic innate immunity; as Zhang [14] reported, mtDNA released into circulation by shock could activate the neutrophils p38 MAPK signal pathway via TLR9 and potentially contribute to the development of post-traumatic SIRS. In this study, we also demonstrated that the plasma mtDNA concentration was positively correlated with markers of systemic inflammatory response in terms of NLR. This finding indirectly suggested that mtDNA was involved in the systemic inflammatory response following trauma. Therefore, summarizing all of the abovementioned information, we propose that plasma mtDNA is more powerful as a predictor of post-traumatic inflammatory related complications, including SIRS, than is circulating nuclear DNA.

Moreover, our results demonstrated that plasma mtDNA concentration on admission had a moderate discriminative power to predict the risk of post-traumatic SIRS, although no better than that of WBC or CRP. The observed specificity (0.76) and sensitivity (0.67) of plasma mtDNA in this current study also presents limitations for clinical use. However, given that there were no statistical correlations between plasma mtDNA and WBC, as well as CRP, this result may indicate that the original sources or the mechanisms implicated in the release of mtDNA are different from those responsible for WBC or CRP; thus, in this sense, the measurement of circulating plasma mtDNA may complement WBC and CRP in the prediction of post-traumatic SIRS. Conversely, our results indirectly indicated that the increased change in the plasma mtDNA was observed earlier than changes in the WBC. Therefore, although the predictive power of plasma mtDNA is less favorable than that of WBC, it would be more appropriate to use plasma mtDNA to predict post-traumatic SIRS soon after trauma, potentially allowing preventive anti-inflammatory interventions, as well as the improvement of survival in patients with post-traumatic SIRS.

Although the current study demonstrated that a higher circulating plasma mtDNA concentration on admission is an independent predictor for post-traumatic SIRS, this study still has several limitations, as follows: First, we must clarify that the healthy control group was not age- or sex-matched with the study population and that the plasma mtDNA concentration might be influenced by these two factors. Second, the number of patients enrolled in this present preliminary study was limited, specifically in the post-traumatic SIRS present subgroup. Third, we only collected one blood sample from each patient on admission for plasma mtDNA assessment, and we were unable to review the dynamic changes in the plasma mtDNA, which would provide additional valuable information on the prediction of post-traumatic complications. Moreover, the study populations received heterogeneous treatments based on the preference of their doctors. This variation in treatment may have led to potential bias in the statistical analysis. Further large-scale prospective studies with a greater number of patients are required for a better evaluation of the predictive contribution of plasma mtDNA to post-traumatic complications.

## Conclusions

The plasma mtDNA level significantly increases after trauma and independently correlates with the clinical severity of trauma patients. Additionally, the median plasma mtDNA concentration was significantly higher in the patients who developed post-traumatic SIRS compared with the patients who did not. Moreover, the plasma mtDNA on admission was an independent indictor with moderate discriminative power to predict the risk of post-traumatic SIRS, although this discriminative power was no better than that of WBC or CRP. However, further large-scale prospective studies with more patients are required to demonstrate the reliability and reproducibility of plasma mtDNA in predicting post-traumatic complications.
